# Experimental study on the mechanical properties of Guiyang red clay considering the meso micro damage mechanism and stress path

**DOI:** 10.1038/s41598-020-72465-x

**Published:** 2020-10-15

**Authors:** Yanzhao Zhang, Shuangying Zuo, Rita Yi Man Li, Yunchuan Mo, Guosheng Yang, Min Zhang

**Affiliations:** 1 College of Resource and Environmental Engineering, Guizhou University, Huaxi New Campus, Guiyang, 550025 China; 2 Liyang City Construction Development Group Co., Ltd., Liyang, 213300 China; 3 HKSYU Real Estate and Economics Research Lab, Hong Kong Shue Yan University, North Point, Hong Kong; 4 Civil Engineering and Architecture College of Wuyi University, Wuyishan, 354300 Fujian China

**Keywords:** Solid Earth sciences, Engineering, Materials science

## Abstract

This study investigated the macroscopic physical and mechanical properties of Guiyang red clay during surcharge loading, lateral excavation and lateral unloading with axial loading, and clarified the failure mechanism of microstructure before and after shear under different stress paths of *CTC*, *RTC* and *TC*. Consolidated undrained triaxial shear permeability, *SEM* scanning, *XRF* fluorescence spectrum analysis and *XRD* diffraction tests were conducted to simulate the actual engineering conditions. The stress–strain curve, shear strength, pore water pressure variation rule and macroscopic failure mode of soil samples under different stress paths were analysed. In addition, Image Pro Plus 6.0 and *PCAS* were used to study the relationship between the macro mechanical properties and micro microstructure failure under different stress paths. The stress–strain curves from *CTC*, *RTC* and *TC* in *CU* tests were different, with the peak values of shear stress under the three stress paths being *P*-increasing, equal *P*-path and *P*-decreasing path. Moreover, the internal friction angle and cohesion of the increasing *P* path were higher than those of equal *P* path and decreasing *P* path, hence, the influence of stress paths on the cohesion is greater than that of internal friction angle. The pore water pressure is strongly dependent on the stress path, and the variation characteristics of pore water pressure are consistent with the change in the law of the stress–strain curve. Under the same confining pressure in the *P*-increasing path, the shear failure zone runs through the whole soil sample, and the shear failure zone is significant, whereas under the condition of the *P*-reducing path, the shear failure angle of soil sample is about 65°, 55° and 45°, and in the equal *P* path, the soil sample is dominated by the confining pressure, with no obvious microcrack on the surface of the soil sample. The difference is that the distribution of pores in the path of increasing *P* and equal *P* is directional, and the anisotropy rate is small, while the distribution of pores in soil samples with shear failure and before shear is random and the anisotropy rate is high.

## Introduction

Red clay is brownish yellow or brownish red slope wash and residual soil slope in which carbonate rocks form under humid conditions after weathering and laterisation^1^. The study area is located in subtropical monsoon moist climate and karst landform, with highly developed karstification and very significant laterisation in the later period, resulting in a very special soil-forming process, gradually cementing the loose dissolution residual deposits from the new oxides to form the soil structure connection. Finally, the structure connection promotes the loose materials to become massive soil, which improves the loose soil structure. The resistance of the residual deposits is eroded, and the soil is dyed red by iron oxide, with minerals invading the soil, decomposing and recombining to form red clay^2^. Therefore, the red clay in Guiyang is formed by physical and chemical weathering, laterisation, and eluviation of carbonate rocks in an environment of high temperature, humidity, and abundant rainfall. It is brown red and brown yellow in colour, with characteristics such as high liquid limit, high plasticity, high void ratio, low compressibility, obvious irreversibility of expansion and contraction, and ‘reverse section’^3^. In Guiyang red clay, flaky clay minerals, a small amount of acicular dispersed minerals and micro amorphous materials are contacted, connected, and arranged according to certain distribution rules. Moreover, a special meso–micro-structure morphology is formed through cementation, sedimentary environment, and various geological forces acting after deposition.

Numerous studies and practices have proven that the engineering properties of soil are closely related to the meso–micro-structure (such as particle distribution and physical composition), the mechanical properties of loading shear tests under the three stress paths of *P* (*σ*_*1*_ increased, *σ*_*3*_ decreased) are completed totally different, where *σ*_*1*_ is the maximum principal stress, *σ*_*3*_is the minimum principal stress, and *P* is ((*σ*_*1*_ + *σ*_*3*_)/2). Different loading paths also play a controlling role, such as increasing *P* (*σ*_*1*_ increased, *σ*_*3*_ unchanged) and decreasing *P* (*σ*_*1*_ unchanged, *σ*_*3*_ decreased). Therefore, it is of great significance to consider the influence of the micro-damage mechanism and stress path on the mechanical properties of soil. Since Terzaghi, people have begun to realise the dependence of the static and dynamic characteristics of soil on the soil structure and the stress path, which has attracted the attention of many scholars^4^. Lambe et al.^5,6^ first proposed the concept of stress path, elaborated the specific research method of stress path, provided reasonable reference for the application field and test of soil, and detailed how to consider the influence of stress path in engineering. However, the systematic study of the deformation, strength, and stress–strain curve of soil by the stress path is still immature. Nagaraj et al.^7^, Vaid et al.^8^, Kumruzzaman and Yin^9^, and Ng and Chiu^10^ have conducted a variety of experimental studies on soil stress paths according to the developed new loading test device. Although the new test device is a good test method for the research of the soil stress path, in most cases, the research and development of the instrument is only aimed at a specific soil, which has strong regionality and high cost and is difficult to promote. Therefore, it is necessary to study a large number of stress paths based on ‘general test instruments’, Bakasubramanian et al.^11^, Lade and Duncan^12^ drew the stress path of an undrained test. According to the similarity of the effective stress path, consolidation pressure could be used. At the same time, an experimental study on sand showed that when the initial stress state and the final stress state are the same, different stress paths correspond to different stress–strain curves. In addition, Huang Zihong et al.^13^, Burland et al.^14^, Shen et al.^15^ and Liu and Shen^16^ studied the mechanical properties, strength, and destructiveness of red clay and structural soil with different stress paths through a large number of tests and theoretical analyses. As Terzaghi^17^ pointed out, the structure of soil has a considerable influence on its mechanical properties. Jia et al.^18^, Hu et al.^19^ and A.N., Usov et al.^20^ respectively considered the influence of different disturbance degrees, different deformation stages, and different loading methods on the soil microstructure. Through the consolidation compression test, SEM, and particle analysis test of soft soil, the microstructure of the soft interlayer (soft soil) and cohesive soil in Shanghai under the accumulated macro-deformation of soil was studied. However, the evolution mechanism was mostly confined to a static state, and the analysis of the evolution process of continuous change was insufficient. Therefore, Lin et al.^21^, Zhang et al.^22^, Chen et al.^23^ and Zeng^24^, respectively, conducted a cyclic load test, repeated shear test, and a triaxial shear test on Hangzhou soft clay, loess and mudstone, alkali-polluted red clay and South China soft soil, and using the SEM scanning test and PCAS rock and soil structure identification and quantitative analysis system, the researchers studied the soil microstructure composition, revealing the characteristics and combination rules of the directional distribution of soil particles and pore space, but the above research objects are mostly for the soft soil or loess in the coastal area, less for the red clay, and the most adopted methods are relatively simple. There is a lack of research on the stress path, micro-mechanism, and control mechanism of soil. In addition, Ng et al.^25^, Castelblanco et al.^26^, Tang et al.^27^, You et al.^28^, Xu et al.^29–31^ studied other special soil masses and analysed the micro-mechanism under macro-mechanics.

The above research results based on the macro- and micro-laboratory tests provide the basis for understanding the mechanical properties and micro-action mechanism of soil but do not consider the change in the mechanical properties of the original soil structure after the reorganisation and failure, or clarify the impact of the micro-structure on the macro-mechanics. Further analysis showed that the findings of the above research method are only a kind of ‘fitting’ reflected by the soil structure, which cannot explain the mechanical properties of the soil essence, and it is more difficult to master the basic factors that determine the soil properties, and thus, it is difficult to accurately predict the engineering properties. Therefore, it is very difficult to accurately describe the stress characteristics of soil under different stress paths and reveal the law of stress variation under the influence of various factors. This paper discusses the macro-mechanical properties of Guiyang red clay under different stress paths and confining pressures. The purpose of this study was to explore the correlation between the different macro-mechanical properties of laterite in Guiyang and the change in its microstructure. Through the analysis of its microstructure, we could study its engineering properties, understand the engineering properties of laterite, and study its evolution law, which provided a theoretical basis for the study of the mechanical properties of the soil in the study area and the failure of the relevant engineering structures of laterite.

## Materials and methods

### Material preparation

The test soil samples were taken from the engineering geophysical exploration test site area (106°39′30.16″, 26°26′38.40″) of the College of Resources and Environmental Engineering, Guizhou University, with a sampling depth of 4–7 m. The soil samples were formed by the dolomite of the Anshun formation of the Triassic system through various weathering processes. They were brownish red with a small amount of brownish yellow, plastic-consistence plastic state.

### Material properties

**Figure 1:**
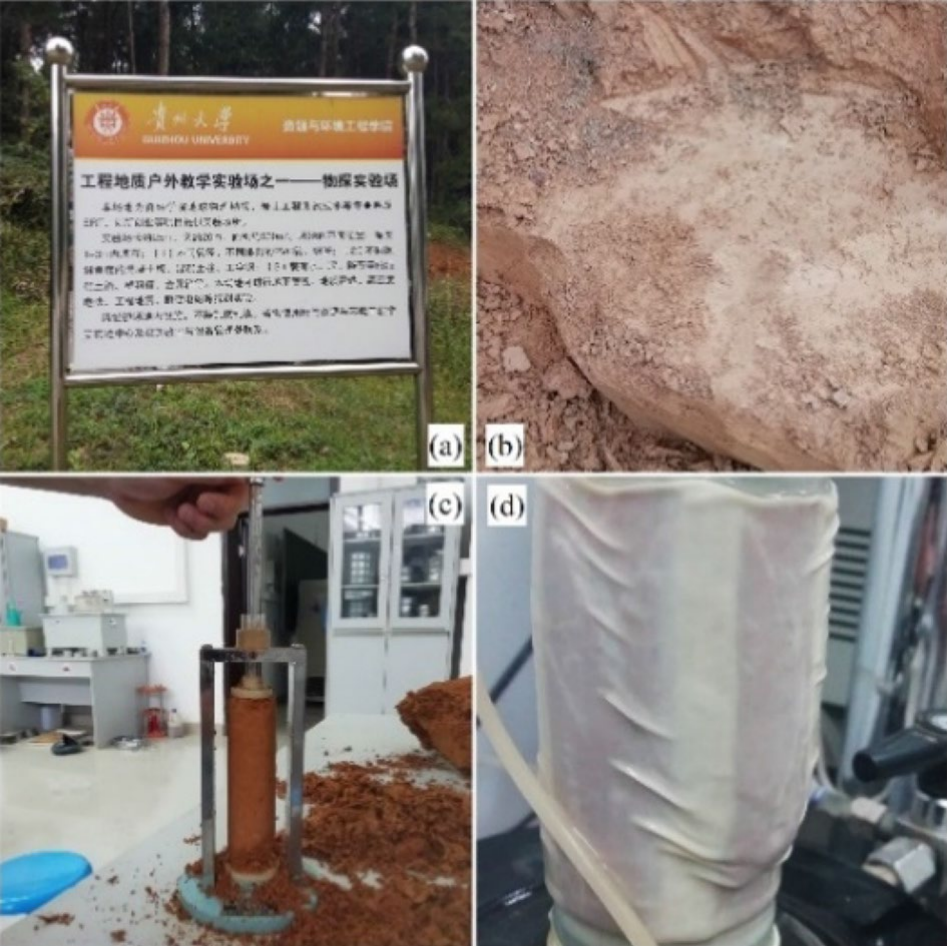
The appearance characteristics of land points and test sample^34^. (**a**) Land points; (**b**) field soil samples; (**c**) test sample; (**d**) soil sample in experiment.

**Figure 2:**
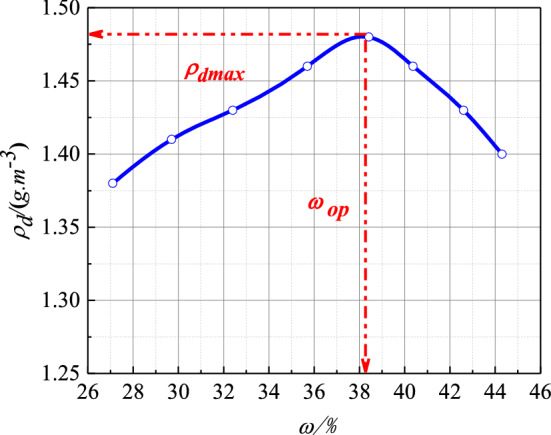
Compaction curve of red clay from Guiyang.

The soil was defined as hard plastic clay as per ASTM, D2487^35^. In this study, the soil was obtained from under the ground. The natural moisture content and the dry density of the red clay was 42.50% and 144 g/cm^3^, respectively. The basic physical parameters are shown in Table 1, and the chemical components of red clay are shown in Table 2. The characteristics of land points and test sample are shown in Fig. 1. The red clay from Guiyang was subjected to a standard compaction test as per ASTM D698-12^36^, and the compaction curve is shown in Fig. 2. It can be seen from Fig. 2 that the optimum moisture content of red clay was 38.41%, while the maximum dry density was 1.48 g/cm^3^.

**Table 1:** Physical parameters of red clay.

Dry density (g/m^3^)	Moisture content (%)	Liquid limit^a^ (%)	Plastic limit^a^ (%)	Porosity ratio	Specific gravity^b^
1.44	42.50	69.21	41.55	1.23	2.63

^a^As per ASTM D4318-10^32^; ^b^as per ASTM D854-10^33^.

**Table 2:** Chemical components of red clay.

Components	SiO_2_	Al_2_O_3_	Fe_2_O_3_	TiO_2_	K_2_O	SO_3_
Content (%)	58.84	23.96	9.36	2.60	2.00	1.69

**Figure 3:**
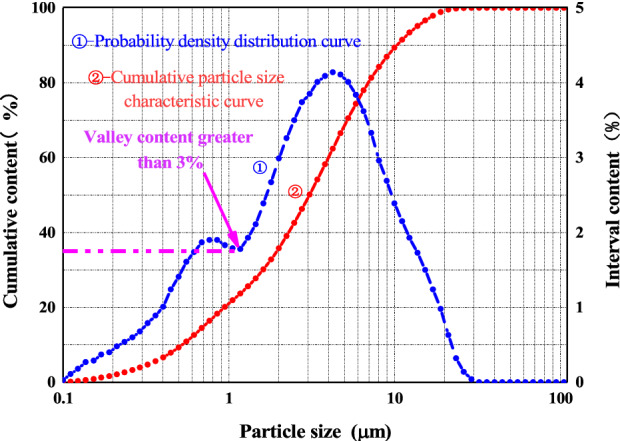
Compaction curve of red clay from Guiyang. The Y-coordinate is the percentage of the particle mass smaller than a certain particle size. For the probability curve of particle size, the X-coordinate is the average particle size of each particle group and the corresponding soil content is shown in Y-coordinate^38^.

According to ASTM D422-63^37^, the particle size distribution of red clay was determined with a laser particle size analyser. Figure 3 shows the probability curve of the particle size and the cumulative particle size distribution curve of Guiyang red clay. Curve ① in Fig. 3 shows a double peak. The soil content at the two peaks was 39.04% and 83.26% respectively, while in the valley, the point between the two peaks was 37.89%. This was attributed to the fact that the study area was dominated by the carbonate strata, consisting of calcite, dolomite, and a small amount of non-carbonate rocks and insoluble impurities such as clay minerals. Under the simultaneous existence, mutual promotion, dissolution, deposition, and metasomatism, the destruction of the original parent rock structure was relatively complete. The dissolution residue no longer had the skeleton of the parent rock structure, which was loose and porous sand. Non-gravel, massive weathered debris and lamellar flakes during karstification considerably changed the proportion of the mineral composition of the eroded residual deposits. The dominant soluble minerals in the parent rock dissolved and were carried away with the current. The insoluble impurities with little content were enriched and became the main component of the deposits, and the dissolving residual deposits were further evolved after red clayification to form red clay. Therefore, as a sediment mixture, Guiyang red clay had a dual granular structure with two peaks: the lower peak was a clay system mainly composed of flaky clay minerals, containing a small amount of granular sand and amorphous materials; the higher peak was carbon. The acid rock formed a semi-weathered zone of weathered crust, the residual bedding structure of the original rock, and an aggregate containing more cemented substances such as iron oxide and aluminium oxide. Therefore, even if the probability curve of the particle size distribution had two peaks and the soil gradation was discontinuous because the valley point value between the two peaks was far greater than 3%; this further indicated that the red clay still had good gradation.

**Figure 4:**
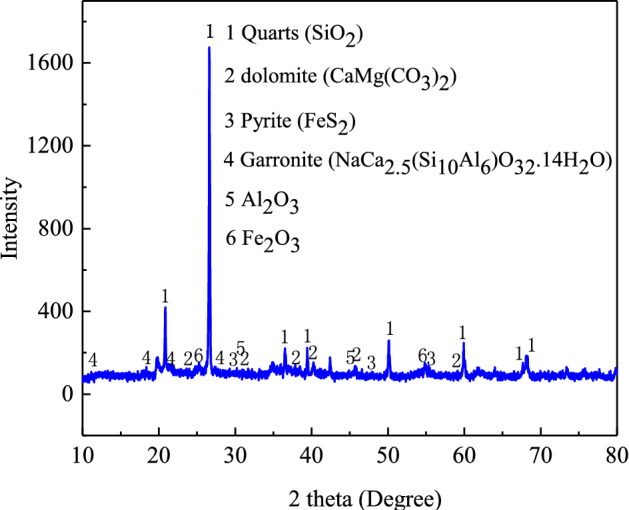
XRD diagram of red clay from Guiyang.

From Fig. 4 and Table 2, the X-ray diffraction (*XRD*) diagram showed that Guiyang red clay mainly contained quartz, pyrite, dolomite, dolomite, and cruciform zeolite. These minerals were the primary minerals consisting of montmorillonite or chlorite, illite, and kaolinite in clay minerals. The pyrite played a certain role in cementing the soil particles. Moreover, because of the existence of pyrite, the colour of the soil was usually red.

### Detection method

#### Triaxial test scheme (CU test)

**Figure 5:**
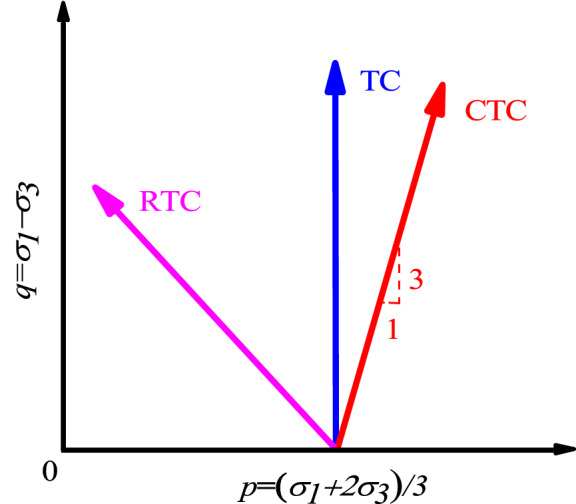
Diagram of loading with different stress paths^40^.

The *CU* tests was performed as per ASTM D2850^39^ on the original soil sample, φ 39.1 mm × 80 mm. First, areas of the field with better soil samples were selected and the remaining soil around the selected area was removed using a shovel and pick, ensuring a sufficient earth borrowing face. Small geotechnical testing tools were used to cut out the larger test soil sample, which was then manually processed with a wire saw to obtain the test soil sample (39.1 mm × 80 mm). Finally, the soil sample was wrapped in multilayer plastic wrap to prevent water loss and the samples were completely sealed with melted paraffin. For this experiment, 27 samples were prepared and randomly divided into three groups, with nine samples per group. The top and bottom of the soil sample were covered with soaked filter paper, with eight wet filter paper strips evenly attached to the side of the sample, then the film tube was tightened by the rubber film. Finally, the soil sample was fixedly on the triaxial instrument clay plate by the membrane tube and rubber band. In this test, the SLB-1 strain controlled triaxial shear permeability tester, the shear control method was the stress control. For the undisturbed Guiyang red clay samples, a confining pressure of 300 kPa, 400 kPa and 500 kPa was applied to drain and consolidate the samples (isobaric consolidation). Then, under the condition of no drainage, triaxial shear tests of the three stress paths were conducted, conventional shear (*CTC*, ${\upsigma }_{1}$*σ*_***1***_ increased, ***σ***_***3***_ unchanged) in the process of surcharge loading, reduced shear (*RTC*, ***σ***_***3***_ decreased, ***σ***_***1***_ unchanged) in the process of lateral excavation unloading and compression shear (*TC*, ***σ***_***3***_ decreased, ***σ***_***1***_ increased) in the process of lateral unloading (Fig. 5). The specific test plan is shown in Table 3.

**Table 3:** Different stress path loading schemes (*CU* test).

Stress path	Consolidation method	Consolidation process (kPa)		*K*_*0*_ value	Shearing process	Shear rate
		Consolidation confining pressure final value				
		*P* = (*σ*_*1*_ + 2*σ*_*3*_)/3	*q* = *σ*_*1*_ − *σ*_*3*_			
*CTC*	Isobaric consolidation	300	0	*k*_*0 *_= 1	∆*σ*_*3*_ = 0; ∆*σ*_*1*_ > 0	∆*q* = 0.6 kPa/min
		400				
		500				
*TC*		300			∆*σ*_*3*_ < 0; ∆*σ*_*1*_ > 0	∆*q* = 0.6 kPa/min
		400				
		500				
*RTC*		300			∆*σ*_*3*_ < 0; ∆*σ*_*1*_ = 0	∆*q* = 0.6 kPa/min
		400				
		500				

According to Table 1 and Formula (1), the saturation of the sample was 90.87%, and the test needs to be conducted in a saturated state, so the sample is saturated. In this paper, the air extraction saturation method was used for saturation. The sample weight was recorded before the sample was placed into the saturator. A thin layer of petroleum jelly was applied between the vacuum cylinder and the cover to ensure a tight cover. The vacuum cylinder was then connected to the exhauster. When the vacuum pressure gauge reading was close to atmospheric pressure (pumping time exceeds 1 h), the pipe clamp was opened slightly to slowly pour clean water into the vacuum cylinder. During the water filling process, the vacuum pressure gauge reading should remain unchanged. Pumping was stopped after the saturator was flooded by water and the pipe clamp was opened to allow air to enter the vacuum cylinder. The vacuum cylinder was then connected to the air extractor, and when the vacuum pressure gauge reading was close to atmospheric pressure (pumping time not less than 1 h), the pipe clamp was opened slightly to allow the vacuum cylinder to slowly fill with clean water. The vacuum pressure gauge reading should remain unchanged during the water injection. After the water flooded the saturator, the pumping was stopped and the sample held for 10 h to become fully saturated. Samples were then removed from the saturator and weighed to calculate the total mass. If the saturation is lower than 95%^41^, it should continue pumping saturation. During the test, the air in the pore pressure measurement pipeline was carefully removed and the pipeline discharged to prevent external air from entering the soil sample. The target confining pressure value was set in the consolidation stage and the pore water pressure under the consolidation termination condition was 0. The sampling was performed at 30 s intervals and different equal confining pressures were applied to complete the drainage consolidation of red clay. To apply shear, the drain value was closed and the pore pressure, axial displacement and axial principal stress difference were manually cleared, the test target value and parameters of the low stress state were defined before gradually applying the confining pressure and axial principal stress difference according to the time interval, so that the soil sample was damaged under a certain stress path.

1 $$ S_{r} = \omega_{sr} \times G_{s} /e $$

In formula (1): *S*_*r*_ is the saturation (%) of the sample; *ω*_*sr*_ is the saturated moisture content (%) of the sample; *G*_*s*_ is the specific gravity of soil particles; *e* is the Porosity ratio of the sample.

#### SEM test

To study the morphology of red clay, *SEM* tests were conducted on undisturbed and remoulded red clay. The test soil sample was cut into small pieces of approximately 1 cm^3^ with a thin steel wire while a groove with a depth of approximately 0.5 mm was cut in the middle of the sample. Then, the prepared sample was placed into the freeze dryer and removed after approximately 24 h. The observed surface in this study was a horizontal profile of soil samples, while the used samples were cut along the direction of soil deposition. Before scanning, the soil sample was gently forced apart with the hands from the groove position, and a relatively flat and fresh section was selected for testing. The broken soil sample was sprayed with gold to improve conductivity. Finally, the gold-coated specimen was moved to the specimen holder for SEM testing.

The microstructure and morphology analyses of Guiyang red clay based on *PCAS* rock and soil structure identification, quantitative analysis system, and Imagepro-plus 6.0 software via *SEM*, were conducted under different stress paths before and after shear to assess the connection and failure mechanism among red clay particle units and the change in the particle microstructure and pore distribution characteristics because of the interaction among the particle units and the soil and water interactions.

#### Image processing tools

*PCAS* is specialized software for the identification and quantitative analysis of pore and crack systems. It was applied for the quantitative identification and structural analysis of rock cracks, pores, shale gas pores and mineral particles. In identifying particles and pores, a variety of their images can be imported. Through binarization, irregular points can be automatically removed so that particles and pores can be automatically divided and recognized, with the geometric and statistical parameters exported and the resultant vector images and rose diagrams displayed. Various geometric parameters of all particles and pores, such as their number, area, length, width, directivity, and shape coefficient, are shown in the data table. Through statistics, the particle and pore content (i.e. pore rate), fractal dimension, area probability, distribution index and other statistical parameters can be obtained to achieve quantitative analyses of mineral particles and rock pore systems.

Image Pro Plus (*IPP*) is a state-of-the-art all-32-bit image processing and analysis system developed by Media Cybernetics (USA), which enables the processing of true colour and black-and-white images compatible with standard image formats. It supports internationally popular image boards and digital *CCD* cameras. Image-Pro Plus 6.0 features powerful *2D* and *3D* image processing, enhancement, and analysis functions, with exceptionally rich measurement and customization capabilities. It can also accurately collate parameters of irregular holes in *SEM* images, including their area, circumference, radius, and number to solve problems arising from applying *IPP* to scanning slice images under an electron microscope. *IPP* is a top-class image analysis software package for fluorescence imaging, quality control, material imaging and many other scientific, medical, and industrial applications.

## Results and discussion

### Stress–strain curve of Guiyang red clay under CU test conditions with different stress paths

**Figure 6:**
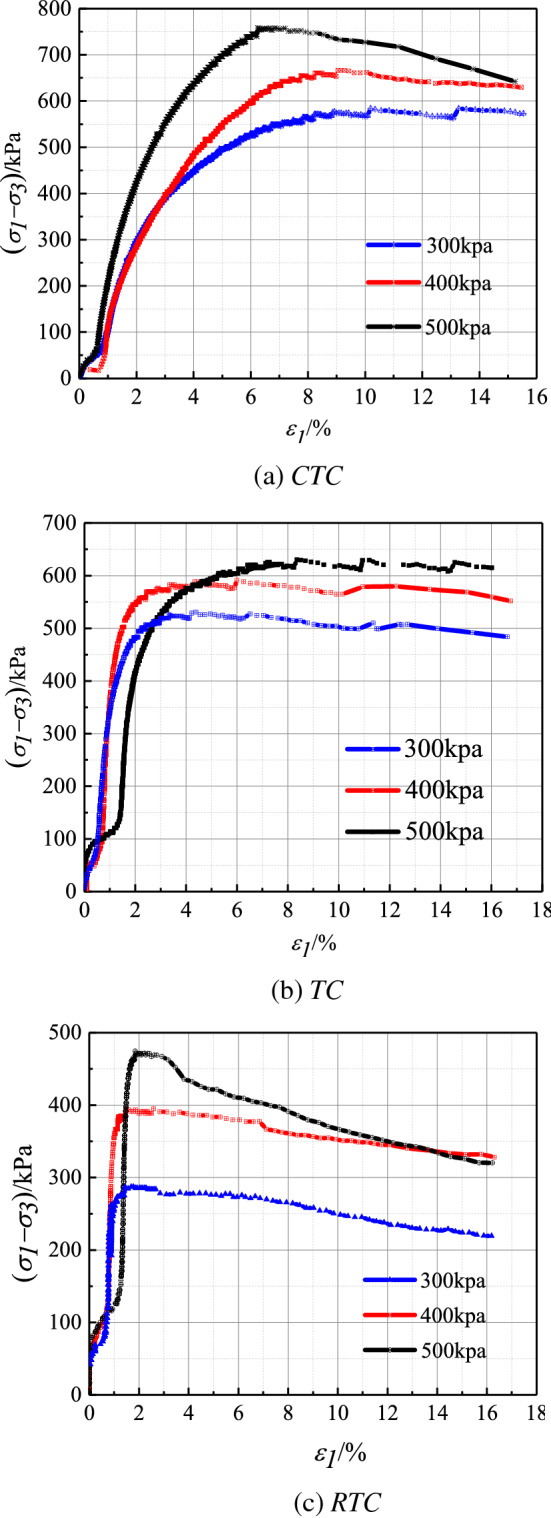
Stress–strain curve of different stress paths of Guiyang red clay (CU test).

According to the standard geotechnical engineering test^41^, the relationship between the axial principal stress difference and the strain is plotted with the principal stress difference as the ordinate and the axial strain as the abscissa. The peak point on the curve is regarded as the failure point and in the absence of a peak value, the main stress difference at 15% of the axial strain is the failure point^41^. Figure 6a–c show the stress–strain curves of red clay under different confining pressures. The preliminary analysis showed that the limit values of the yield stress and strain of the soil structure corresponding to the shear failure of Guiyang red clay under different stresses were different. At the beginning of the load, under the same confining pressure, with an increase in the axial deformation, the main stress difference gradually increased, the initial elastic modulus increased, and the stress–strain curve overall showed a rising trend. In the later stage of the soil yield failure, under the condition of *CTC* loading, the deviator stress was greater than that of *TC* and *RTC*. This showed that in the initial stage of shear failure, the soil structure was damaged and the resistance of the soil itself gradually weakened. At the same time, it was not difficult to see that the undisturbed red clay was easily affected by the loading mode, but the stress–strain curves showed the trend of strain softening. The higher the confining pressure was, the higher were the yield stress and the axial strain of the structure, because of the increased stress, which led to the lateral deformation of soil limited by the confining pressure, slowly compressing the soil sample and thereby increasing the bite force between the red clay particles. Therefore, the pore was compressed, and the required deviation stress under the same strain increased with an increase in the confining pressure.

### Variation characteristics of pore water pressure of Guiyang red clay under CU test conditions with different stress paths

**Figure 7:**
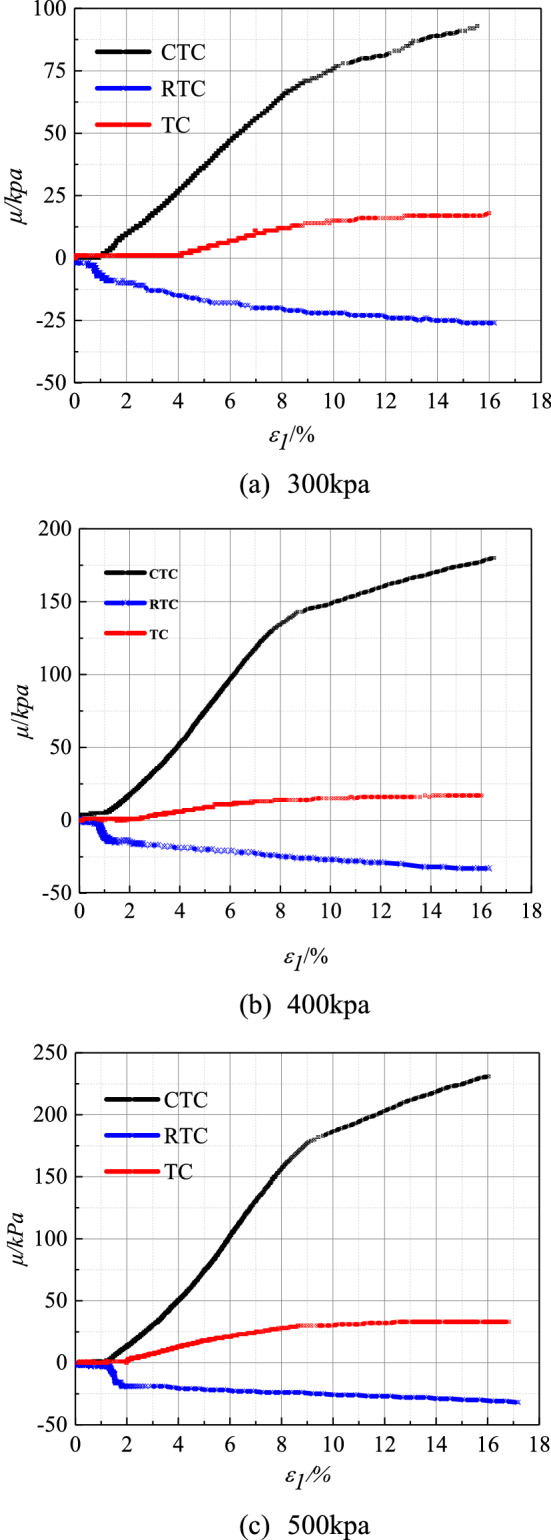
Curve of pore water pressure under different stress paths of Guiyang red clay (*CU* test).

On the basis of the comparative analysis of the pore water pressure curves under different stress paths of Guiyang red clay (Fig. 7), we concluded that the pore water pressure of Guiyang red clay under the same shear path had similar characteristics; that is, under the same stress path, the pore water pressure under different consolidation pressures had the same trend, and the pore water pressure of Guiyang red clay under different shear paths was different. This indicated that the pore water pressure and the stress path had a corresponding relationship, the main difference being that the pore water pressure was larger in the case of the increasing *P* shear path, smaller in the case of the equal *P* shear path, and negative in the case of the decreasing *P*-shear path. Corresponding to the stress–strain curve, when the soil reached plastic deformation failure, the pore water pressure in the path of increasing *P* continued to increase slowly, the pore water pressure in the path of equal *P* remained basically unchanged, while the pore water pressure in the path of decreasing *P* decreased slightly.

In the case of the same stress path, the larger the confining pressure was, the greater was the pore water pressure, indicating that there was a direct relationship between the pore water pressure and the initial consolidation confining pressure. This was most obvious in the case of the *P* increasing stress path. In the shear path of increasing *P*, when the deformation reached approximately 9%, the change rate of the pore water pressure slowed and corresponded to the peak value of the shear stress, with the increased rate of pore water pressure obviously higher than that of the other two stress paths. The change in the pore water pressure of decreasing *P* and equal *P* did not correspond to the curve of the stress–strain relationship, and there was no obvious change in the pore water pressure of the two stress paths and no common point between the two stress paths. The confining pressure decreased gradually during shearing, reducing the constraints around the soil sample under axial compression, leading to the lateral deformation of the soil sample. The continuous increase in the pore water pressure with respect to the smaller pore pressure was the main reason for the decrease in or the instability of the foundation-bearing capacity. Therefore, this potential engineering issue must be considered in actual engineering. In the *P*-shear path, the initial pore water pressure showed a small negative value, but with increased stress, the pore pressure gradually increased to a positive value, and the deformation reached approximately 5% and tended to be gentle. The pore water pressure appeared to be negative, and the author believed that there was a slight expansion effect in the test soil, which led to the negative value of the pore pressure. With an increase in the axial stress in the later period, shear expansion appeared; when the image disappeared, the pore water pressure changed to a positive value. However, in the case of the shear path of reduced *P*, the pore water pressure was always negative, mainly because of the negative pore water pressure formed by the dilatation of soil under the condition of undrained shear, indicating that the negative pore water pressure occurred when there was shear disturbance and the pore ratio increased. It is generally believed that the shear expansion of red clay is caused by the elastic expansion of the over-consolidated sample soil during shear reduction.

**Figure 8:**
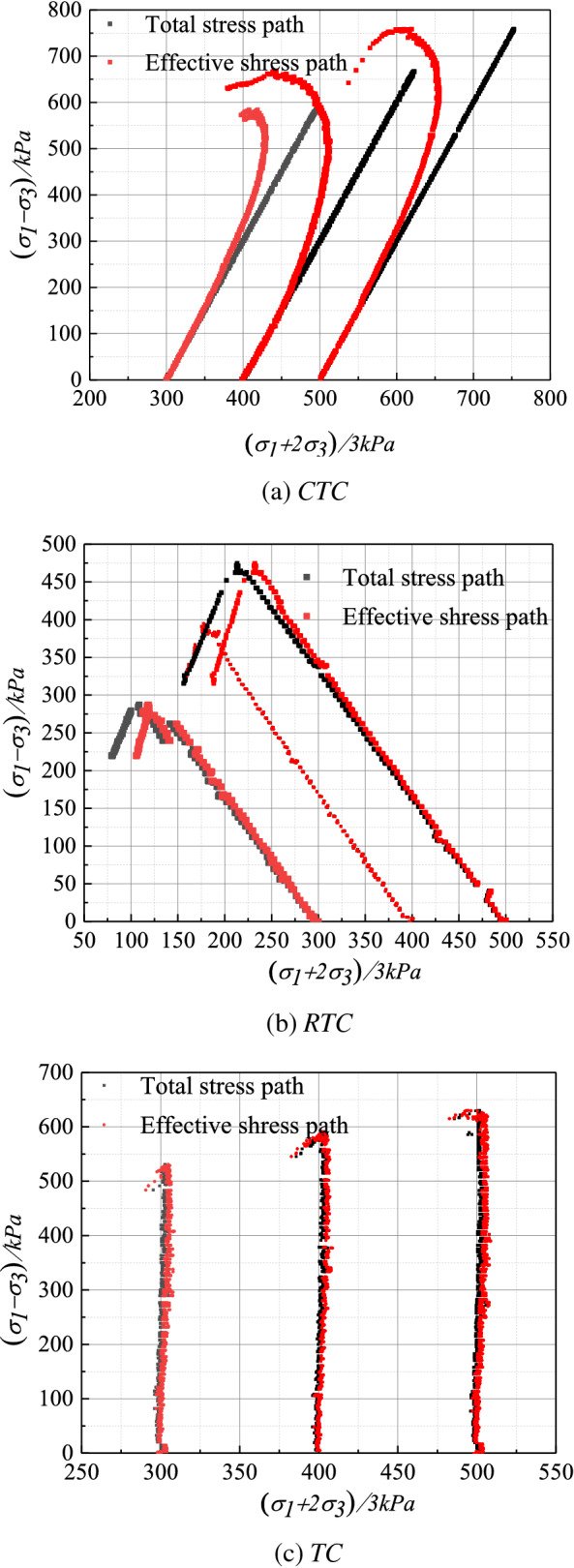
Stress–strain curve of different stress paths of Guiyang red clay (CU test).

Figure 8 is a diagram of the stress path relationship in the *p–q* coordinate system under the condition of isobaric consolidation of Guiyang red clay without drainage. The study found that: (a) under the same stress path, the soil in the initial stage of elastic deformation loading, the total stress path and the effective stress path change trend was similar, close to coincident, and the *RTC* and *TC* stress path is more obvious than the *CTC* stress path, and later with the shearing process, the difference between the effective stress path and the total stress path under the same stress combination gradually increases, after the soil deformation damage reaches the shaping damage stage, the hole water pressure is greatest. Corresponding to the effective stress path under the same shear stress, the total stress difference value reached the maximum; (b) the *CTC* stress path and *TC* stress path, the static pore water pressure in the shear process is greater than zero, the effective stress path curve is more than the total stress path curve to the left, the static gap water pressure under the RTC stress path and the shear process is less than zero, the effective stress path curve is less than the total stress path curve right; (c) The strain softening phenomenon under a high circumference (400 kPa, 500 kPa) is more obvious, and the peak strength *q* is more evident in the stress path diagram, and after the peak, the *p* and *q* values gradually decreases, while the phenomenon is not observed in the stress path of *RTC* and *TC*.

### Shear strength parameters of Guiyang red clay under Cu test conditions with different stress paths

**Figure 9:**
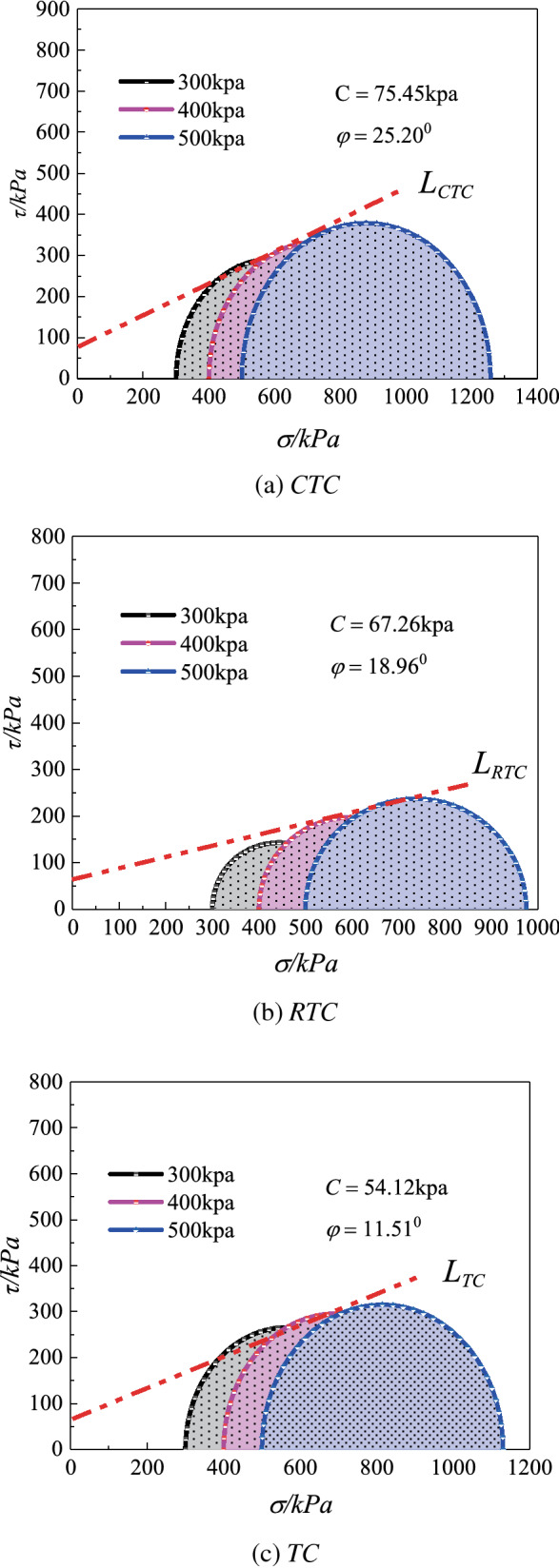
Mohr circle of stress under different stress paths (*CU* test).

According to the standard geotechnical engineering test method^41^, the ordinate was the shear stress, the abscissa was the normal stress, the abscissa axis was (*σ*_*1f.*_ − σ_*3f.*_)/2 $({\upsigma }_{1\text{f}}-{\upsigma }_{3\text{f}})/2$ $({\upsigma }_{1\text{f}}-{\upsigma }_{3\text{f}})/2$ $({\upsigma }_{1\text{f}}-{\upsigma }_{3\text{f}})/2$ $({\upsigma }_{1\text{f}}-{\upsigma }_{3\text{f}})/2$, the radius was ($({\upsigma }_{1\text{f}}-{\upsigma }_{3\text{f}})/2$ *σ*_*1f.*_* — σ*_*3f.*_)/2, the damage stress circle was drawn on the stress plane *σ—τ*, and the common tangent of each circle was drawn under different confining pressures, in which the common tangent was the cohesion and the inclination was the internal friction angle (as shown in Fig. 9).

The shear strength parameters of the undisturbed red clay in the cases of the three stress paths of increasing *P*, decreasing *P*, and equal *P* are listed in Table 4, wherein for the undisturbed red clay, the internal friction angle and cohesion of the red clay itself gradually decreased because of the remoulding effect of the three stress combinations of increasing *P*, decreasing *P*, and equal *P* on the soil structure. The specific performance was that the shear strength of the red clay under the condition of increasing *P* was greater than that in the case of decreasing *P*, and that in the case of the decreasing *P* was greater than the performance in the case of equal *P*. The change in the shear strength of the soil was influenced to a certain extent by two factors. First, the mineral composition of the soil itself, the level of particles, and the structural characteristics of the soil, which are congenital factors, determined the basic physical and mechanical properties of the soil, which made the deformation and the failure mode complicated and diversified. Second, the existence of the load effect on the soil led to the remoulding of the soil structure, which was obviously manifested in the compaction of soil particles and the change in the pore water pressure. The results showed that the internal friction angle and the cohesive force of red clay were the largest under the condition of increasing *P*, which made it more difficult to produce shear failure than the conditions of decreasing *P* and equal *P*, followed by the condition of decreasing *P*, which occurred relatively easily. This phenomenon was attributed to the effect of the different pore water pressures on the soil samples, the strength difference of the confining pressure on the soil samples, and the common effect of the axial pressure on the soil samples in the cases of the three stress paths. Therefore, in the engineering practice of Guiyang red clay, it was necessary to comprehensively analyse the internal friction angle of the soil and the relationship between the cohesion and the stress.

**Table 4:** Shear strength parameters of Guiyang red clay under different stress paths (*CU* test).

Parameter value	*CTC*	*RTC*	*TC*
Internal friction angle (°)	25.20	18.96	11.51
Cohesion (kPa)	75.45	67.26	54.12

### Macro failure mode of Guiyang red clay under Cu test conditions with different stress paths

**Figure 10:**
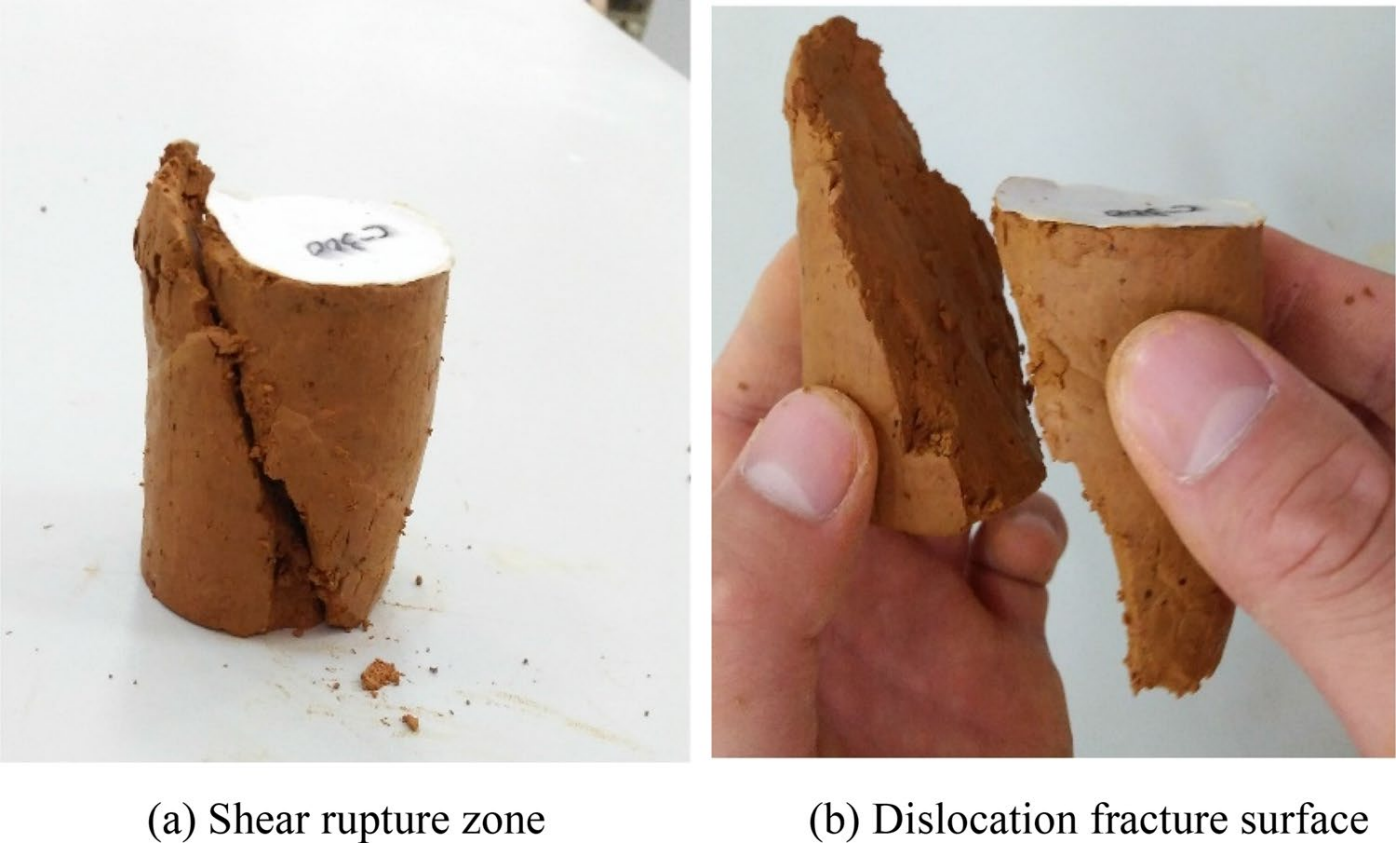
The visual characteristics of the land points and test sample.

On the basis of the stacking process of different stress combinations and the test results, we concluded that the shear failure of Guiyang red clay was a gradual process from local to overall development, and the shear failure was gradually completed, which was described as a form of a shear band during the shear process. Figure 10 shows the typical macro-failure mode of Guiyang red clay, with a large shear failure zone and soil disturbance. The failure surface and the horizontal plane were approximately 60°, with some micro tension cracks appearing near the shear zone under the stress of the upper soil body. Micro-cracks developed to a certain extent, and the strain rate of the soil body continued to increase. The soil body structure was seriously damaged, and the upper soil body followed the shear fracture zone, which gradually penetrated, leading to dislocation and detachment of soil. The size of the soil particles, distribution of the pore size, anisotropy of the structure, and other microstructure parameters change as a result.

**Figure 11:**
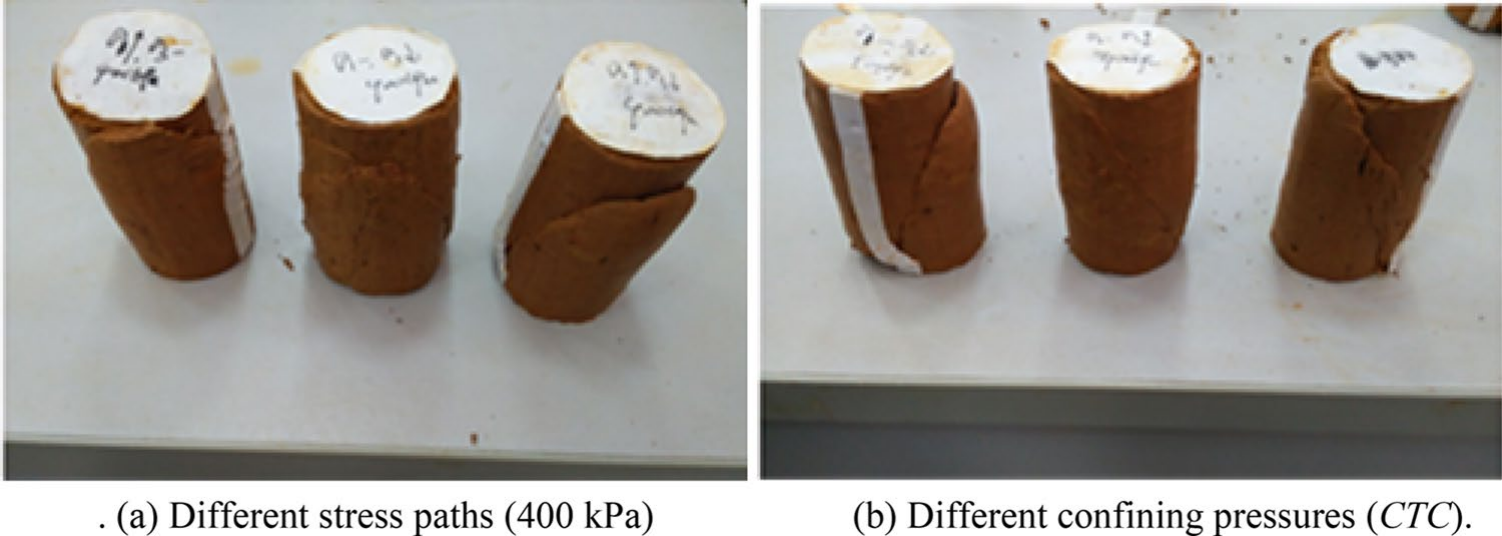
Macroscopic failure modes of samples under different stress paths and different confining pressures of Guiyang red clay.

Figure 11a shows the shear failure of soil samples with different forms in the case of different stress paths. At the same confining pressure, under the condition of increasing *P*, the shear failure zone ran through the entire soil sample, and the shear failure zone was significant, whereas in the case of decreasing *P*, the soil mass had an expansion contraction effect, and the surface tension crack was large, with an obvious shear failure zone. Under the condition of equal *P*, the soil sample was dominated by the surrounding pressure, and there was no obvious microcrack on the surface of the soil sample, except in the shear failure zone.

In Fig. 11b, at different confining pressures, the shear failure of the soil samples having the same stress path also had different forms. Taking the conventional stress combination as an example, when the confining pressure was 300 kPa, 400 kPa, and 500 kPa, the shear failure angle of the soil sample was approximately 65°, 55° and 45°, respectively, and there was an inverse correlation between the confining pressure and the shear failure angle. In general, there was a close relationship between the macro-change in the shear failure of Guiyang natural red clay and the application of an external load.

**Figure 12:**
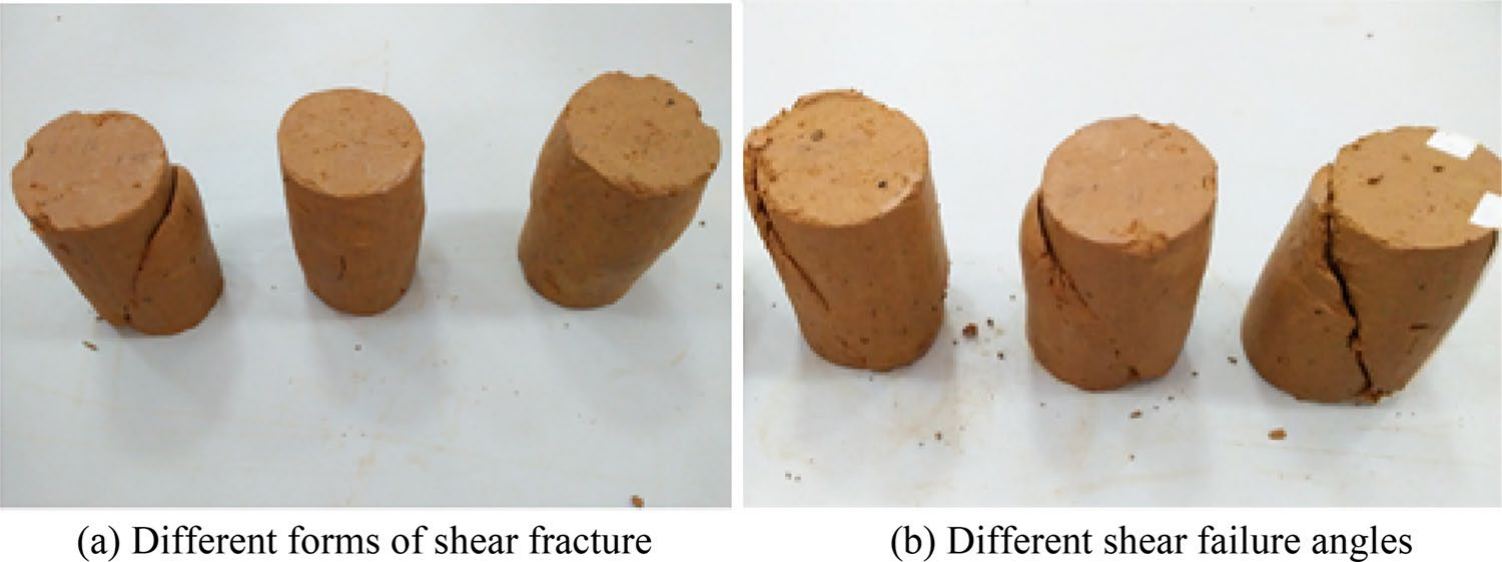
Macroscopic failure modes of different stress paths of Guiyang red clay under *CU* test conditions.

The combination of the same stress led to the formation of a variety of soil structures in red clay. Along with the slow shear of the soil, the shape of the macro broken ring near the shear failure zone of the soil sample intensified, gradually forming a cross shear failure zone with different shapes. The results showed that the failure modes of the *CU* test of Guiyang red clay in the case of different stress paths could be divided into shear and swelling. As shown in Fig. 12a, the shear failure was caused by the increase in the constant axial pressure of the confining pressure, the tension crack was caused by the decrease in the constant axial pressure of the confining pressure, and the bulging deformation had no obvious failure surface. Most samples formed a shear failure band with obvious penetration during the shear process, and the shear failure angle was generally within the range of 45°–60°, accompanied by some local micro-cracks, as shown in Fig. 12b.

## Microstructure analysis of Guiyang red clay under Cu test conditions

### Fine microstructure characteristics of red clay under different stress paths

**Figure 13:**
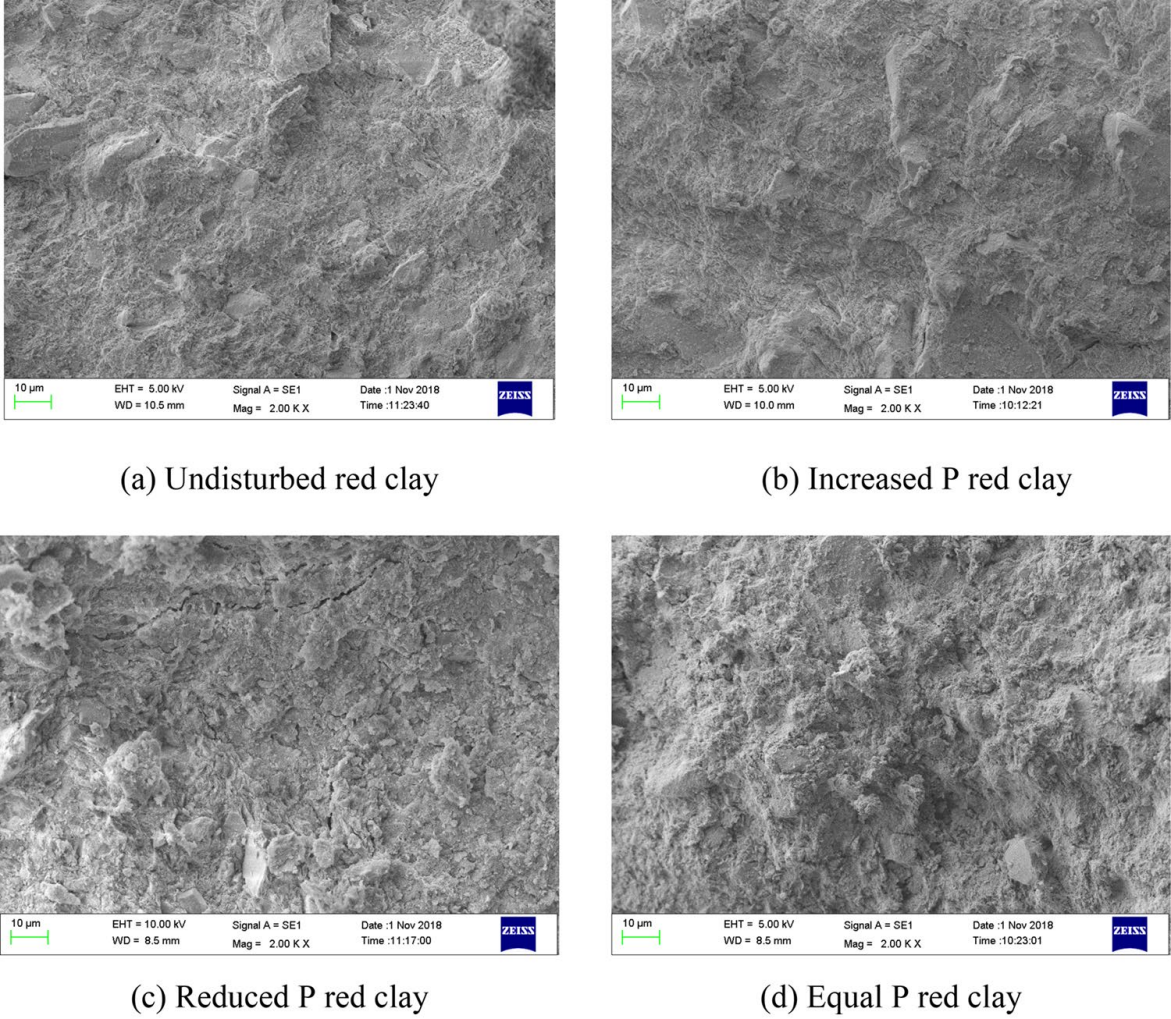
SEM images of Guiyang red clay.

**Figure 14:**
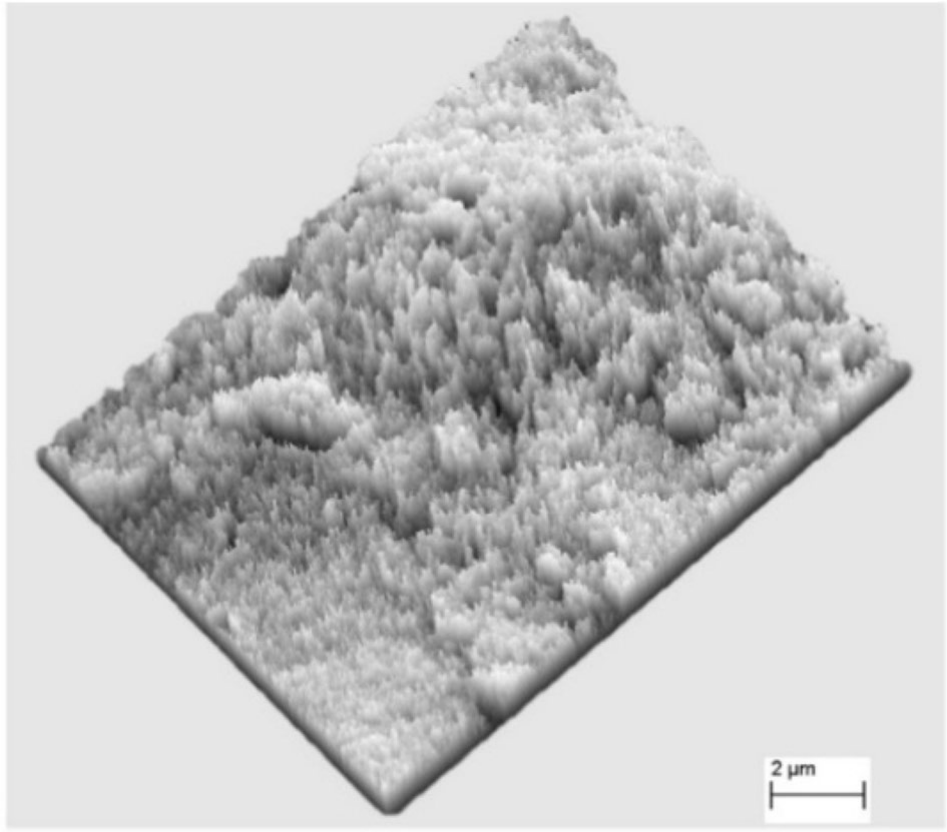
Three-dimensional visualisation of the microstructure of Guiyang red clay.

Regarding the morphological characteristics of the microstructure of Guiyang red clay in different stress paths, the microstructure elements of red clay were integrated into the shape and arrangement of particles, the shape and arrangement of pores, and the form of cementation^42–44^. The SEM scanning image of the original sample microstructure of Guiyang red clay before and after shearing is shown in Fig. 13. Figure 13a shows the undisturbed sample before shearing, and Fig. 13b–d show the original sample after shearing in the cases of the stress paths of increasing *P*, decreasing *P*, and equal *P*. Before shearing, the structure of undisturbed red clay was relatively loose, with narrow and long gaps, and relatively few pores. There were granular, flaky, and flocculating structures with different scales in the soil mass, among which the characteristics of clay particles were relatively obvious. The contact mode between the soil particles was mostly point to surface contact, surface to surface contact, and point to point contact, with an irregular particle sequence. The consolidation and compaction effect were not obvious in the soil mass, with a small degree of homogenisation of the soil surface morphology, showing anisotropy, and the cementation effect between the soil particles was significant. A three-dimensional visualisation of the microstructure of Guiyang red clay allowed further analysis of the morphological characteristics of the pores, showing that the arrangement, scale shape, and distribution range of the undisturbed red clay pores were irregular and diverse, as shown in Fig. 14. The structural form of red clay after shear failure in the cases of different stress paths was compared and analysed. Driven by external forces, the undisturbed soil samples under the three conditions were compacted to different degrees, the shape characteristics of clay particles were not obvious, the soil flocculation structure was not prominent, and the soil samples had massive agglomerated and flaky structures, lacking pores, rubber, and the nodal effect. The soil particles were loose and disordered in the path of increasing *P*, with more granular groups forming a poorly cemented aggregate skeleton and disturbed and damaged floc structure and pseudo-sphere structure, whereas in the paths of decreasing *P* and equal *P*, the surface of the soil sample was dense and the original structure was seriously damaged. At the same time, the three stress paths made the surface of the soil sample appear different with smooth friction surfaces, with a more obvious effect in the cases of the reduced *P* and the equal *P* stress paths. Considering that the different combinations of stress forced the soil structure to change, the deformation and failure of the soil from the macro aspect was essentially the changes in the internal particles, pores, and cement forms.

### Quantitative analysis of pore structure of red clay under different stress paths

**Figure 15:**
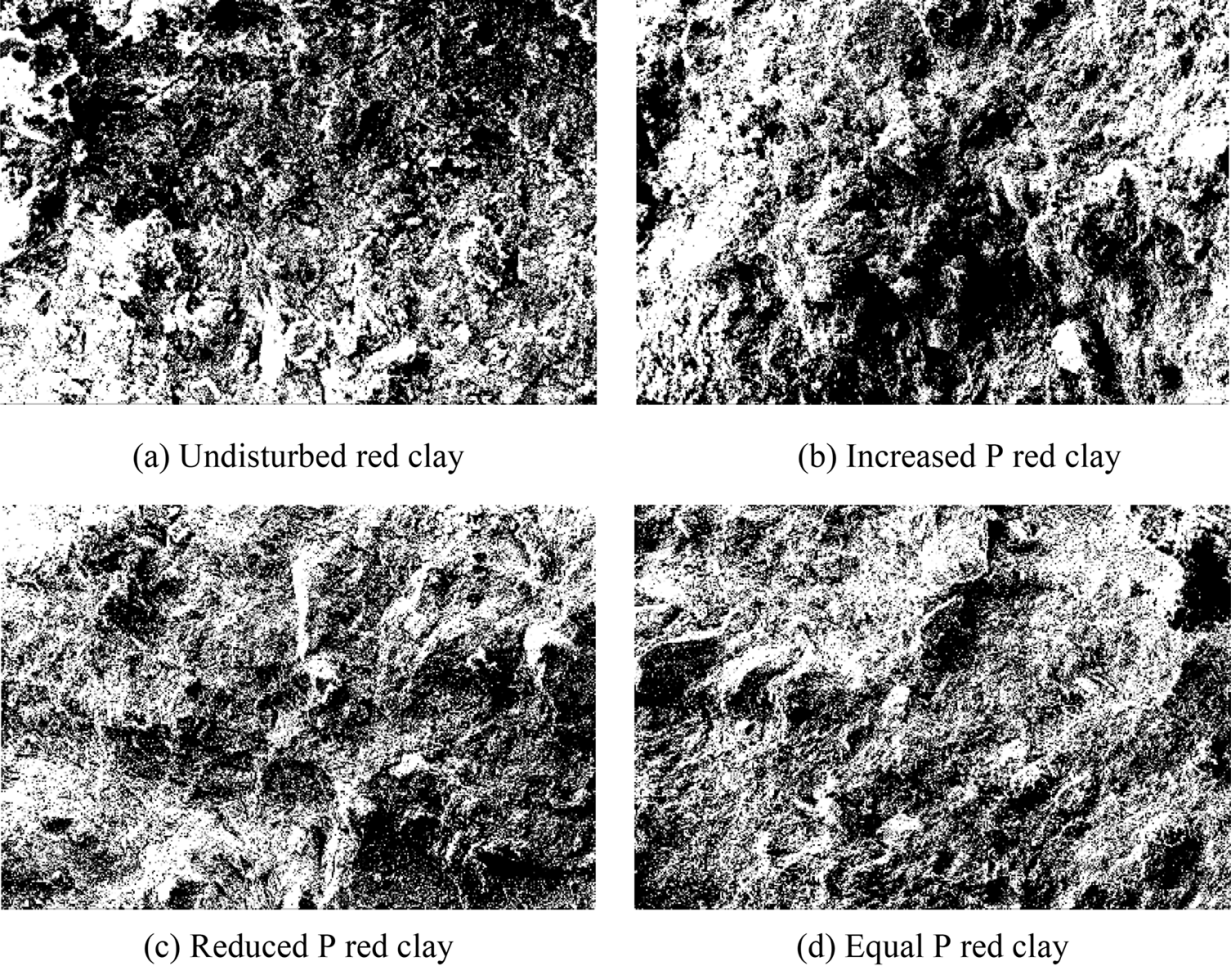
Binarisation results of SEM images of Guiyang red clay. Note: white is soil particle structure, black is soil pore.

**Figure 16:**
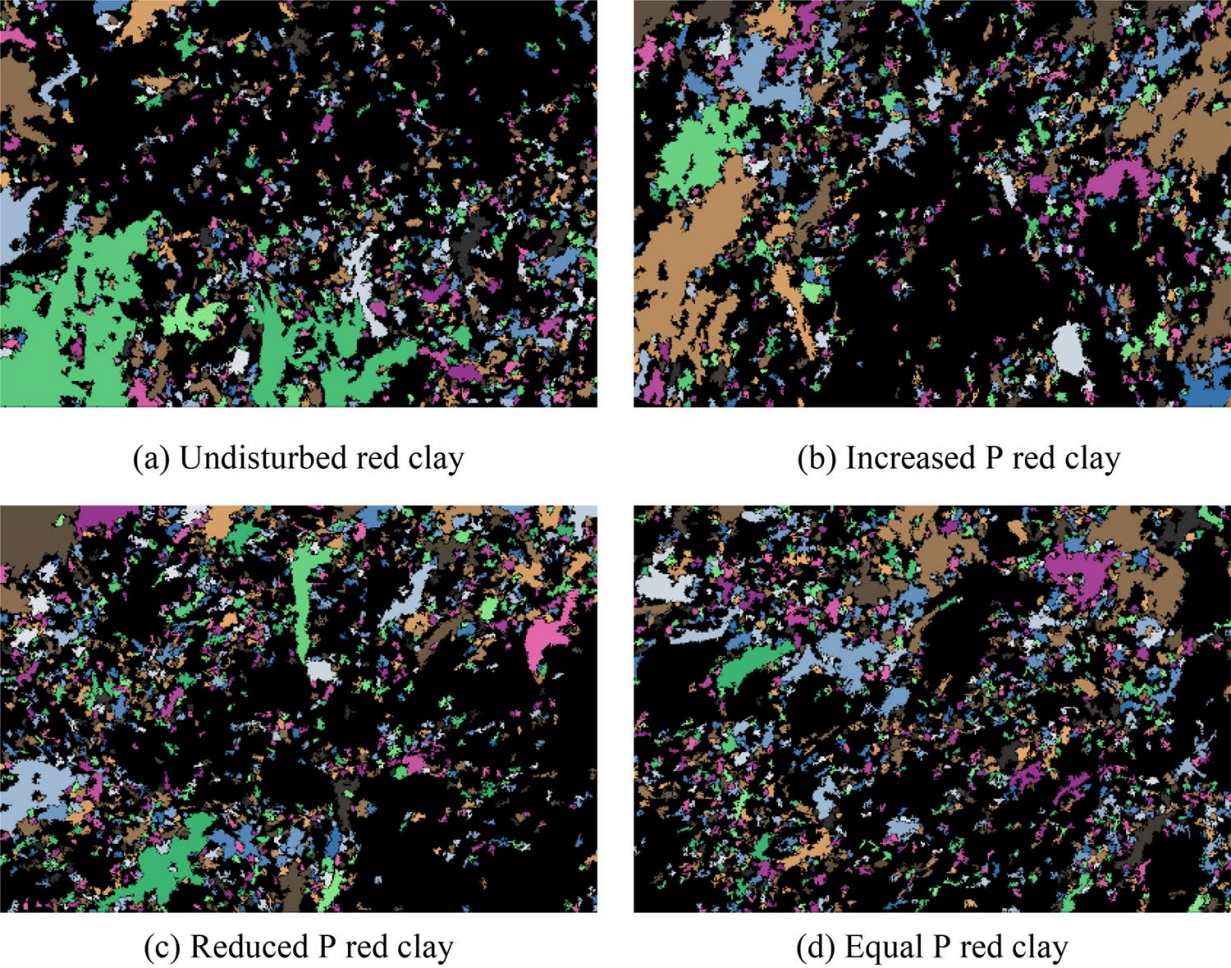
Vectorisation results of SEM images of Guiyang red clay. Note: white and colour are soil particle structure, black is soil pores.

**Figure 17:**
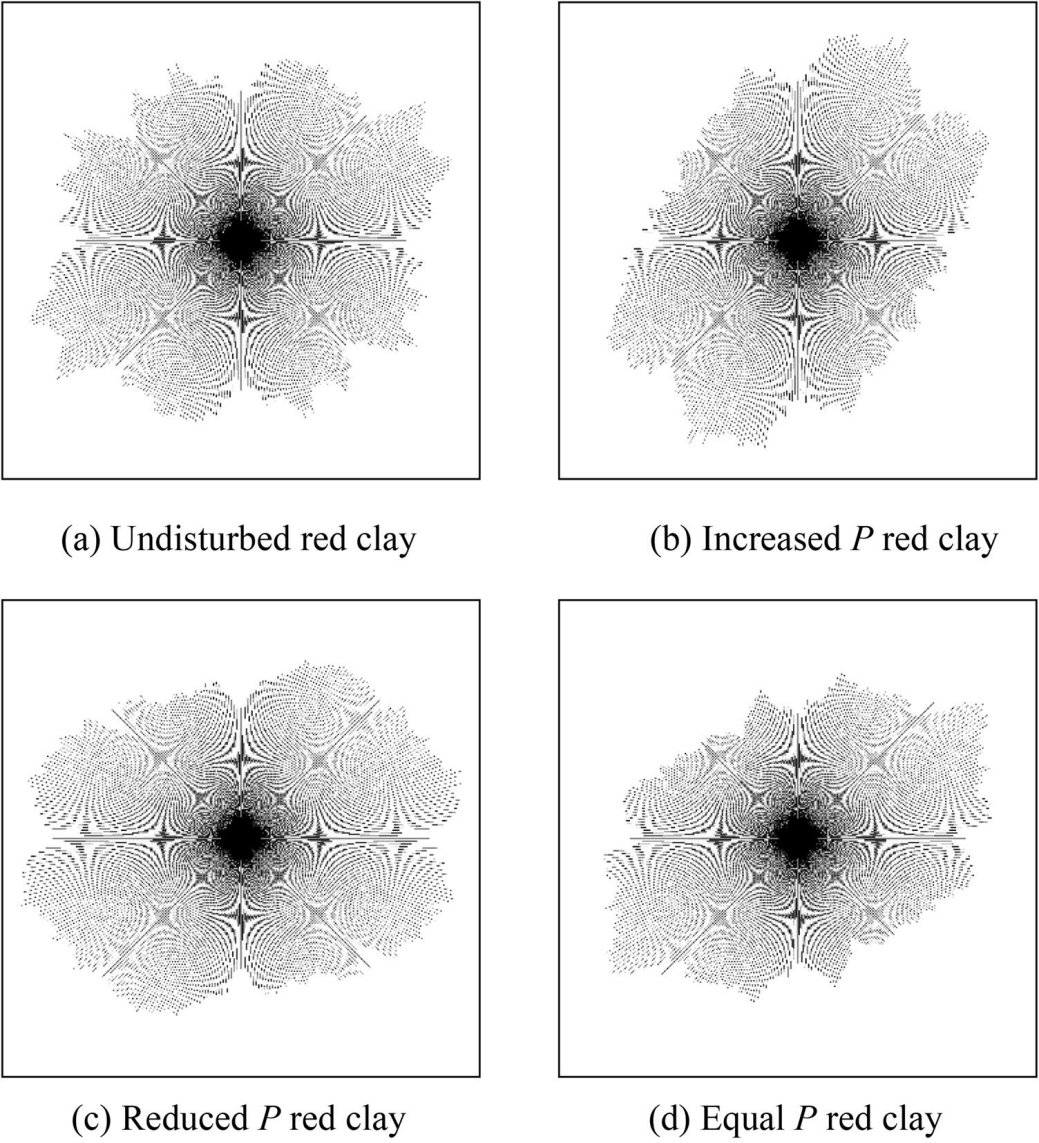
Quantitative treatment of rose distribution in Guiyang red clay.

On the basis of the *SEM* scanning images of Guiyang red clay before and after shearing (as shown in Fig. 13), the binary processing of undisturbed samples for different stress paths was conducted using *PCAS* (as shown in Figs. 15 and 16), in which (a) shows the processing diagram of the undisturbed samples, and (b), (c), and (d) show the processing diagram of the undisturbed samples after shear failure under the action of increasing *P*, decreasing *P*, and equal *P*, respectively. The pore distribution in the path of increasing *P* was more uneven than that in the undisturbed soil with fewer macropores. The irregular arrangement and combination of micro groups and aggregates in the red clay formed an overhead structure. The pores in the path of decreasing *P* and equal *P* were mainly mesopores, and the macropores and micro pores were fewer than those in the undisturbed soil, with close contact between soil particles. At this time, the soil skeleton bore the effect of external stress, with massive and flaky soil particle structures under different stress paths, which might be affected by the stress combination, which compacted the soil particles to form a cohesive soil block, indicating that the soil had entered the shear failure state, and the soil skeleton began to bear the external stress. When we continued to apply the pressure, the soil sample underwent shear failure. According to the distribution of the black area before and after shearing, combined with the pore distribution map of the quantitative pore treatment of Guiyang red clay shown in Fig. 17, the shear failure of the undisturbed soil sample before shearing and Guiyang red clay under the conditions of increasing *P*, decreasing *P*, and equal *P* was compared and analysed. During the shear failure of the soil body under the action of increasing *P* and equal *P*, the pore distribution in the soil body had a certain directionality and anisotropy rate^45^. However, the pore distribution of the undisturbed soil samples before shear and after the *P* reduction had no obvious directionality, with a random distribution of pores and a very low occurrence of anisotropy.

Considering that the undisturbed sample maintained relatively original pore distribution characteristics, while the increase path of *P* was mainly subject to a constant confining pressure and a gradual increase in axial stress process, the soil sample was difficult to shear and damage; therefore, the internal structure of the soil sample was mainly compressive shear, and the disturbance to the soil sample pore was small. Under the *P*-path, the undisturbed soil was mainly affected by the constant axial stress, while the confining pressure gradually decreased. At this time, the lateral constraint of the soil sample decreased, accompanied by axial compaction and lateral shear creep, with a large disturbance to the soil sample. In the case of the *P*-path, the undisturbed soil sample was mainly affected by the gradual decrease in the confining pressure and the gradual increase in the axial stress; therefore, the disturbance was the most serious, and the pores were small and fine. The *PCAS* rock and soil structure identification and quantitative analysis system was used to quantitatively analyse the pore morphology of the SEM scanning images of Guiyang red clay, providing the parameters of the pore morphology of red clay before shear and after shear failure for the different stress paths, as shown in Table 5. According to the change in the weight of the large pore area of Guiyang red clay before and after shearing, the normalised strength of Guiyang red clay before shearing was lower than that of the *P*-reduced stress path, while the normalised strength of Guiyang red clay before shearing was higher than that of the *P*-increased and equal *P* stress path. At the same time, the experimental data showed that the fractal dimension of the undisturbed samples before shear was smaller than that of red clay after the shear failure in the *P*-increasing, *P*-decreasing and *P* -equal tests, indicating that the shear failure of Guiyang red clay occurred in the cases of the different stress paths, the proportion of holes decreased, and the complexity of the hole structure increased, while the agglomeration degree of the soil particles decreased and the shape of holes became irregular, which was the most obvious phenomenon. The shear failure of red clay under the condition of equal *P* was followed by increasing *P* and decreasing *P*.

**Table 5:** Statistical parameters of porosity of red clay in Guiyang.

Stress state	Maximum area porosity	Porosity (%)	Average area (pixel)	Maximum length (pixel)	Average width (pixel)	Fractal dimension	Probability entropy	Regional probability distribution index
Undisturbed sample	47,606	32.91	233.8	432.0	21.3	1.26	0.99	2.36
*CTC*	31,700	36.87	267.3	398.2	13.8	1.28	0.98	2.13
*RTC*	12,196	31.96	175.7	262.0	12.6	1.27	0.99	2.37
*RC*	12,671	33.00	170.2	215.6	21.2	1.31	0.98	2.27

**Figure 18:**
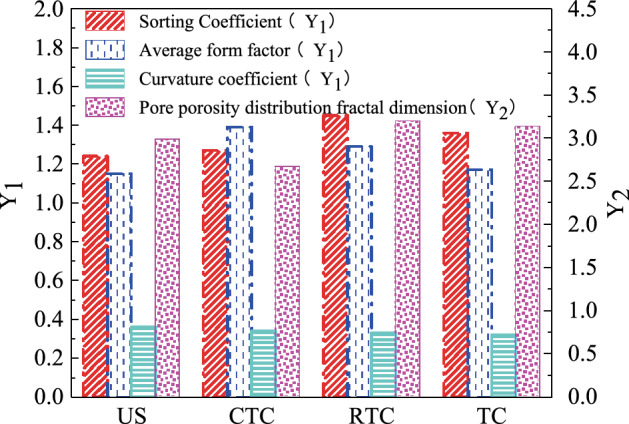
Guiyang red clay pore form histogram.

The deformation of the soil structure caused by different combinations of forces has always been a common phenomenon in engineering, causing numerous engineering problems. However, the deformation of soil is closely related to the changes in the pore shape and scale. Considering the effect of different stress combinations on soil pores, the *PCAS* system was used to quantitatively analyse the changes in the pore shape and size before and after the shear failure of Guiyang red clay, and a double Y-axis coordinate system was established on the basis of the sorting coefficient, curvature coefficient, average shape coefficient, and fractal dimension of the porosity distribution in the quantitative parameters of pores, as shown in Fig. 18. The test data show that the pore sorting coefficient and curvature coefficient of Guiyang red clay after shear failure in the cases of the different stress paths were greater than before the shear failure, indicating that the shear failure of Guiyang red clay under the three stress combinations resulted in the poor sorting of pores and the convergence of macropores. However, in the cases of the three stress paths, the average shape coefficient of the shear failure of Guiyang red clay was smaller than that before the shear failure, indicating that the pores became longer and narrower because of the different combinations of stress before shear, among which the equal *P* stress path had the greatest impact on the pore shape because of the closure of the large pores under the lateral and vertical pressure associated with the small pores. The change in the fractal dimension of the porosity distribution compared with the original sample before shearing decreased in the case of the path of increasing *P* stress, indicating that the uniformity of the pores increased and the difference between the pore sizes was small. Compared with the path of decreasing *P* and equal *P* stress, the fractal dimension of the porosity distribution increased, with the decreasing uniformity of the pores and the large difference in size between the pores.

## Conclusions

The soil mechanical properties of Guiyang red clay were revealed under different stress paths in the *CU* test. It was of great significance to correctly understand the physical and mechanical properties of Guiyang red clay under different stress paths. It can correctly guide the actual civil engineering construction process such as stacking loading, lateral excavation unloading, and lateral unloading and axial loading. It can ensure the safety and reliability of engineering construction. The research conclusions were as follows:

1. The stress–strain curves of the consolidated undrained shear test of Guiyang red clay under different stress paths were different. In the initial stage of shear failure, the soil structure was destroyed and reshaped through a longer process. The resistance of the soil structure was gradually weakened. The peak value of shear stress was the largest under the path of increasing *P* stress. Under the same stress path, the greater the initial consolidation confining pressure of the soil sample, the greater the peak shear stress. And the peak shear stress of the soil sample increases continuously with the increase of confining pressure.
2. The pore water pressure was the largest under the path of increasing *P*, The negative pore water pressure was generated under the shear path of decreasing *P*, And when the soil reaches plastic deformation failure, The pore water pressure has been slowly increasing under the path of increasing *P*, The pore water pressure has been kept constant under the equal *P* path, The pore water pressure under the path of decreasing *P* was still slightly decreased.
3. The shear strength and internal friction angle of Guiyang red clay were different under different stress paths, The internal friction angle and cohesion of the clay were reduced overall, And from large to small. It was the path of increasing *P*, the path of equal *P*, and the path of decreasing *P*.
4. The Guiyang red clay was destroyed by shearing and bulging under different stress paths in the *CU* test. The shear failure angle was generally within the range of 45°–60°, and some local micro cracks were generated. Under the same confining pressure, the soil sample was penetrated by the shear failure zone under the condition of increasing *P* path; the soil mass has expanded and contracted under the condition of decreasing *P* path. There are no obvious micro-cracks on the soil surface under the condition of equal *P* path.
5. The Guiyang red clay was sheared and damaged under the stress path of *CTC*, *RTC* and *TC* in the *CU* test. The average shape factor becomes smaller, the pore becomes narrow and long, In particular, the influence on the pore morphology was greatest under the equal *P* stress path. The anisotropy of soil pore distribution is reduced under the path of increasing *P* and equal *P*, and the anisotropy was increased in the path of decreasing *P*.

## Abbreviations

CU: Consolidated undrained
CTC: Conventional triaxial compression
US: Undisturbed sample
SEM: Scanning electron microscope
RTC: Reduced triaxial compression
TC: Triaxial compression
TSP: Total stress path
ESP: Effective stress path
